# Changes in body composition revealed by bioelectrical impedance analysis reflect strength and motor performance in myotonic dystrophy type 2

**DOI:** 10.3389/fneur.2024.1451537

**Published:** 2024-12-04

**Authors:** Erica Frezza, Giuseppe Merra, Giulia Greco, Mariangela Goglia, Silvia Seraceno, Laura Boffa, Nicola B. Mercuri, Antonino De Lorenzo, Roberto Massa

**Affiliations:** ^1^Neuromuscular Diseases Unit, Policlinico Tor Vergata, Rome, Italy; ^2^Department of Systems Medicine, Tor Vergata University of Rome, Rome, Italy; ^3^Section of Clinical Nutrition and Nutrigenomics, Department of Biomedicine and Prevention, Tor Vergata University of Rome, Rome, Italy; ^4^School of Specialization in Food Science, Tor Vergata University of Rome, Rome, Italy

**Keywords:** myotonic dystrophy type 2, bioelectrical impedance analysis, body composition, outcome measures, motor function tests, gender, metabolic dysfunctions, strength

## Abstract

**Introduction:**

In myotonic dystrophy type 2 (DM2), metabolic dysfunctions are frequent. Therefore, measurement of muscle mass and body composition by non-invasive methods could help in evaluating disease severity and progression. The aim of our study was to investigate, by means of bioelectrical impedance analysis (BIA), whether DM2 patients have an alteration in their body composition and if this finding correlates with strength and motor performances.

**Methods:**

We obtained anthropometric measures, nutritional data, BIA and blood tests in 18 DM2 patients and correlated them with motor function tests.

**Results:**

The 33% of male and 22% of female patients had BMI values compatible with severe obesity, whereas 44% of males and 33% of females had a metabolic syndrome. Considering BIA parameters, phase angle was under normal values in 56% of males and 89% of females. This may be regarded as a marker of deranged cell membrane function. Motor tests showed a fair to strong direct correlation with several BIA parameters.

**Conclusion:**

These data suggest that BIA findings may be faithful markers of the degree of muscle wasting and impairment in DM2. The different degree of BIA alterations between genders indicate that body composition undergoes sex-related modifications in this disease. The potential of this technique to capture changes in a slowly progressive disorder such as DM2 should be tested in longitudinal studies.

## 1 Introduction

Myotonic dystrophy is an autosomal dominant disease and is one of the most common muscular dystrophies in adult patients. Two types of myotonic dystrophy are known, type 1 (DM1), which is caused by a CTG expansion of the DMPK gene on chromosome 19, and type 2, which is caused by a CCTG expansion in intron 1 of the ZNF9 gene on chromosome 3 (1). Myotonic dystrophy type 2 (DM2) is characterized by myotonia, myalgia and muscle weakness, principally at proximal and axial muscles. Concerning extra-muscular manifestations, posterior sub-capsular cataracts, cardiac conduction defects, insulin-insensitive type 2 diabetes mellitus, and other endocrine abnormalities are the most common (1). A gender-related influence on muscle symptoms and on multisystemic involvement was reported by Montagnese et al. (2) in a retrospective study. At variance with DM1, no evidence of correlation between CCTG repeats and clinical manifestations are reported in DM2 (3).

Although DM1 is relatively common, DM2 is considerably rarer, being tenfold less prevalent than the former in Italy (4). This is a major obstacle when representative cohorts are needed in order to clarify the disease natural history and, therefore, selecting clinically meaningful outcome measures (5). In addition, the relatively non-specific clinical signs of DM2 represent a further complication on the way of establishing sensitive and specific rating scales.

Metabolic alterations are a common feature in DM2 patients: in particular, diabetes type II and insulin insensitivity are reported in around 25–75% of cases, and the incidence increases with advancing age. Also, Renna et al. (6) reported a lower expression of insulin receptor on DM muscle cells and reduced insulin signaling activation that could lead to an imbalance between protein synthesis and degradation. The influence of insulin insensitivity on muscle weakness pathogenesis in DM2 is under debate.

The features of multisystem involvement common to DM1 and DM2 are regarded as characteristic of a progeroid disease. Indeed, sarcopenia of aging and muscle wasting of DM, share several pathogenic mechanisms, including genomic instability, insulin resistance, defective muscle regeneration, imbalance between protein synthesis and degradation, mitochondrial dysfunction and satellite cell senescence (7). Therefore, as reported in aging studies, measurement of muscle mass and body composition by non-invasive methods could be of interest in evaluating disease severity and progression in DM (7).

Bioelectrical impedance analysis (BIA) is a non-invasive, non-expensive, easy to use technology that provides useful information in real time about overall body composition. BIA has been used in several studies of normal and diseased human muscle (8–10), as well as in triplet repeat expansion disorders (11). Indeed, BIA was recently reported to correlate with strength parameters in patients with DM1 (12).

The aim of our study was to investigate, by means of BIA, whether DM2 patients present an alteration in their body composition, whether there are sex-related abnormalities, and if this finding correlates with strength and motor performances.

## 2 Materials and methods

This is a single-center, cross-sectional pilot study of body composition analysis, assessed by BIA, in association with tests of muscle strength and endurance in DM2 patients. Patients with DM2 in regular follow-up at the Neuromuscular Diseases Unit of Policlinico Tor Vergata were offered to participate in this study. The study protocol was approved by the local ethical committee (n.53/21) and designed was conducted in accordance with the Helsinki declaration. All participants gave their written consent before entering the study protocol.

Inclusion criteria were: (a) a genetically confirmed diagnosis of DM2; (b) patient’s accordance to provide informed consent; (c) age between 18 and 80 years. Exclusion criteria were; (a) the inability to complete the purposed questionnaires; (b) to have a pacemaker or an implantable cardiac defibrillator; (c) pregnancy; (d) failure to provide the informed consent.

A group of healthy subjects were recruited as control group by screening consecutive patients at the Department of Clinical Nutrition of the same hospital, according to the same inclusion and exclusion criteria (apart from DM2 diagnosis). Moreover, control subjects had to have normal values of hand grip force, as measured by a hand-held dynamometer (HHD).

The study protocol included BIA, strength and motor measures, questionnaires and nutritional evaluation. Motor evaluation was performed in all subjects by the same neurologist after a specific training. The assessment included the following:

- Quantitative muscle testing (QMT) was performed by a HHD (CITEC, Naarden, The Netherlands) in the following muscle groups: neck flexors, neck extensors, shoulder abductors, elbow flexors and extensors, hip flexors and extensors, knee flexors and extensors.
- Global motor function was assessed through the quick motor function test (QMFT).
- Muscle endurance was tested by the 30s chair to stand test (30CST).

QMT was performed twice per each muscle group on both sides, and the higher value was considered. For each subject, a QMT sum score was calculated, by adding values of muscle groups from both sides (5). The percentage of patients presenting QMT values of single muscle groups under the P5 and P50 normative values were calculated (13). Hip extensor muscles were not considered in this analysis because normative values for this group were not available from the manufacturer. The QMFT is a test composed by 16 items with a score ranging from 0 (poor performance) to 64 (good performance). The 30CST is used to evaluate endurance and motor function of lower extremities. The test starts with the patient seated on a chair with their arms crossed against the chest. The score is the total number of stands achieved within 30s. Each patient was assessed by a physician specialized in nutritional science. The comprehensive evaluation, conducted under standardized conditions, included a detailed medical and nutritional history, physical examination, and specific anthropometric measurements. Body composition was assessed using standardized methods, with procedures consistently repeated for each participant. Waist circumferences (WC) were measured with a professional tape, weight and height were obtained using a professional balance with a built-in stadiometer (SECA 711 Physician’s Balance, Seca^®^), BMI was calculated dividing the body weight by squared height (kg/m^2^), BIA was performed using a single-frequency analyzer (BIA 101S, Akern/RJL Systems, Florence, Italy). Body composition analysis was carried out using Bodygram Plus software (Akern/RJL Systems, Florence, Italy). Calculated parameters included: phase angle (PA), fat mass % (FM), fat mass index (FMI, kg/h^2^), fat-free mass % (FFM), fat-free mass index (FFMI, kg/h^2^), skeletal muscle mass % (SMM), skeletal muscle mass index (SMMI, kg/h^2^), body cell mass % (BCM), and body cell mass index (BCMI, kg/h^2^). All measurements were conducted according to standard guidelines, including patient preparation and consistent execution by a trained operator. All normative values are corrected for age and gender. Blood test included glucose, insulin, vitamin D, total cholesterol, LDL and HDL fraction and triglycerides; homeostasis model assessment (HOMA) was calculated as HOMA-IR = (fasting glycemia [mg/dL] × fasting insulin [mU/L])/405, and insulin resistance was defined if HOMA index was higher than 2.5 (14). The data were analyzed with Prism 7.0 and Excel. Data normality test was assessed with the Shapiro–Wilk test. Descriptive statistics are reported as mean (standard deviation, SD) or median (inter quartile range) according to the distribution of each continuous variables. To analyze differences between groups we used Student’s *t*-test or Mann–Whitney *U* test, accordingly to the variable type. Comparison between categorical variables were made by Fisher’s exact test. Correlation studies were assessed with the Pearson’s correlation test for continuous variables and Spearman’s correlation coefficient for ranked variables. Values of *p* < 0.05, <0.01, and <0.001 were considered significant, highly significant, and extremely significant, respectively.

## 3 Results

### 3.1 Demographic and clinical characteristics

We recruited 18 genetically confirmed DM2 patients, equally distributed between males and females. Healthy controls and DM2 groups did not differ for age, height, weight, and BMI, as shown in Supplementary Table S1. Patients’ demographics are reported in Table 1. No difference in age between males and females was found at Mann Whitney test. Regarding WC, 67% of males and females were above normative values for gender (n.v. <80 cm for women; <94 cm for men) (14). The 33% of male and 22% of female had BMI values compatible with severe obesity. According to International Diabetes Foundation criteria (15), 44% of males and 33% of females were diagnosed with a metabolic syndrome. Insulin resistance, diagnosed by HOMA index value >2.5, and low values of vitamin D were found in 57% and 80% of males and 43% and 71% of females, respectively. Not surprisingly, there was a strong direct correlation between BMI and WC (*r* 0.85, *p* < 0.0001) in DM2 patients, and both BMI and WC showed a direct correlation with HOMA index (BMI *r* 0.72; *p* < 0.01; WC *r* 0.65 *p* = 0.01) and an inverse correlation with serum vitamin D levels (*r* − 0.53 e *p* = 0.05).

**Table 1:** Demographic and clinical characteristics of DM2 patients.

Patient (*n*^b^)	Age^a^	BMI^a^	WC^a^	HOMA index^a^	Metabolic syndrome^b^	Diabetes mellitus II^b^	Hypertension^b^	Dyslipidemia^b^
DM2 (18)	50.3 ± 15	28.5 ± 5	94 ± 12	2.4 ± 1.4	39%	12%	29%	69%
Female (9)	48.8 ± 11	28.1 ± 6	88 ± 12	2.2 ± 1.6	33%	11%	22%	75%
Male (9)	52 ± 18	29 ± 4	100 ± 10	2.7 ± 1.4	44%	13%	38%	63%

BMI, body mass index; WC, waist circumference; DM2, myotonic dystrophy type 2.

a Mean ± SD.

b Percentage of total.

n = number of patients.

### 3.2 Bioelectrical impedance analysis

**Figure 1:**
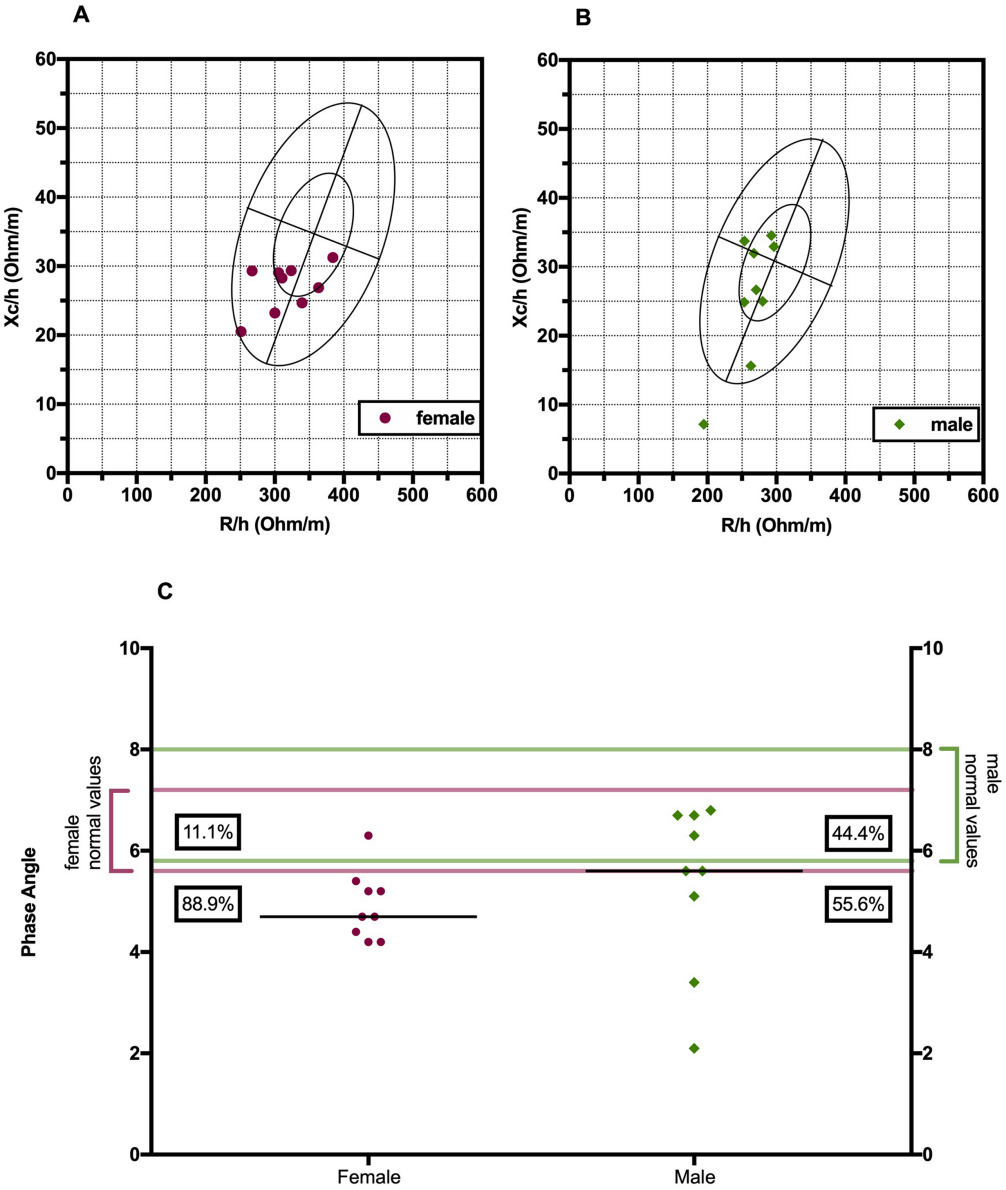
Biavector^®^ in female **(A)** and male **(B)** DM2 patients. The plots display sex-specific tolerance ellipses at the 50 and 95% percentiles. The vertical axis represents hydration status, with higher values at the bottom, and the horizontal axis shows cell mass, with higher values on the left side. **(C)** Distribution of phase angle values of DM2 patients with their corresponding normal range values. H, height; Xc, reactance; R, resistance; PA, phase angle.

**Figure 2:**
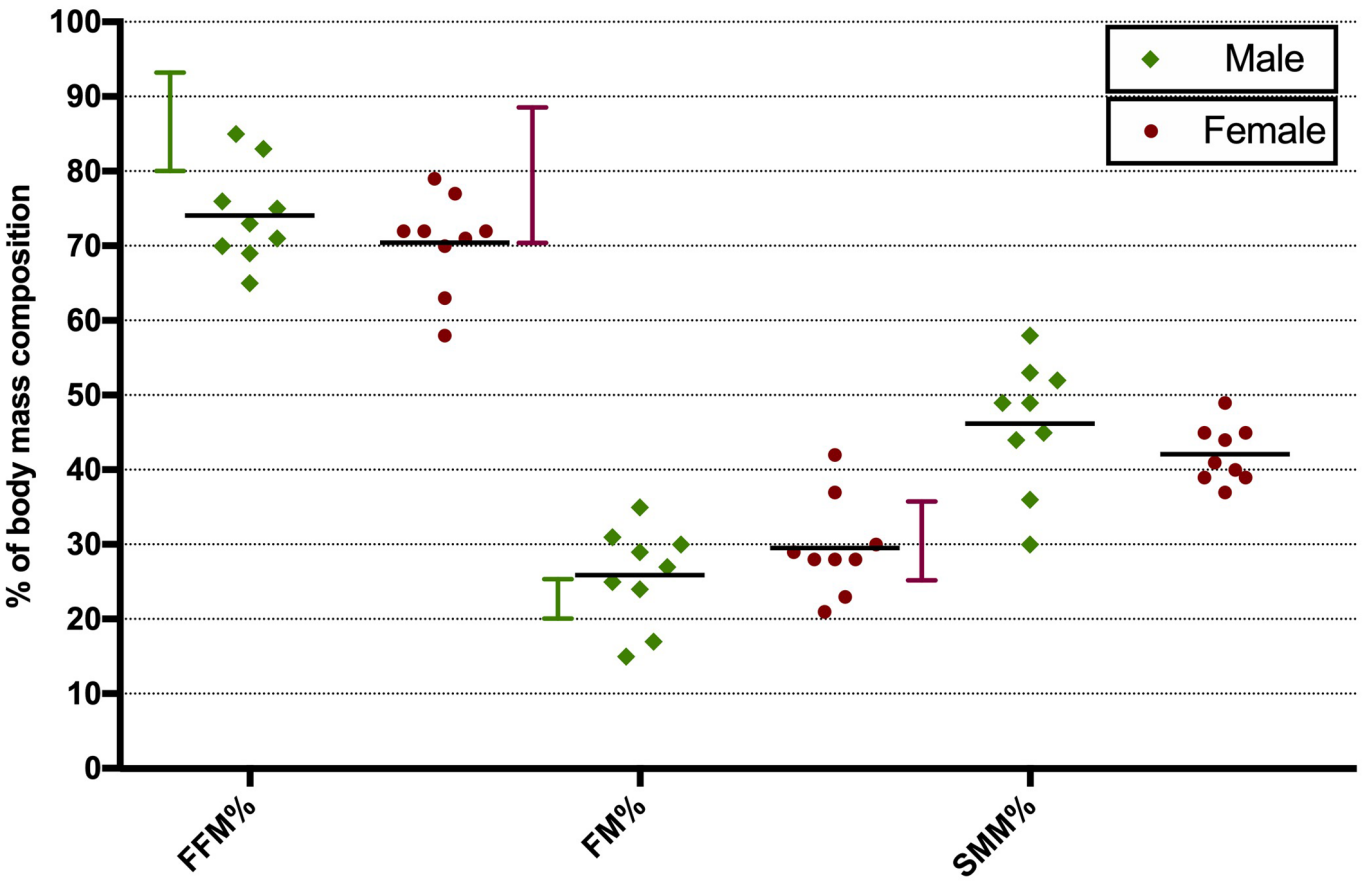
Distribution of BIA parameters in male and female DM2 patients. Vertical bars show normal range values. Horizontal bars show mean values. FFM, fat-free mass; FM, fat mass; SMM, skeletal muscle mass.

**Figure 3:**
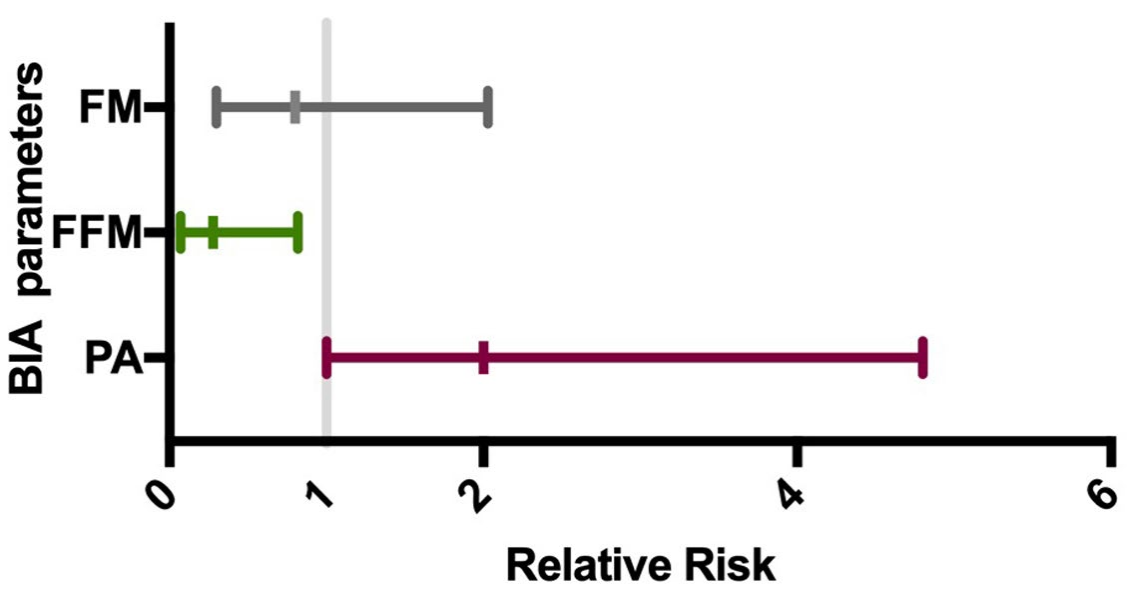
Gender impact on BIA alteration. The forest plot shows the gender relative risk for BIA variables alteration with its 95% confidence interval (horizontal bars). On the left is represented a higher risk for male and on the right for female. FM, fat mass (%); FFM, fat-free mass (%); PA, phase angle.

**Figure 4:**
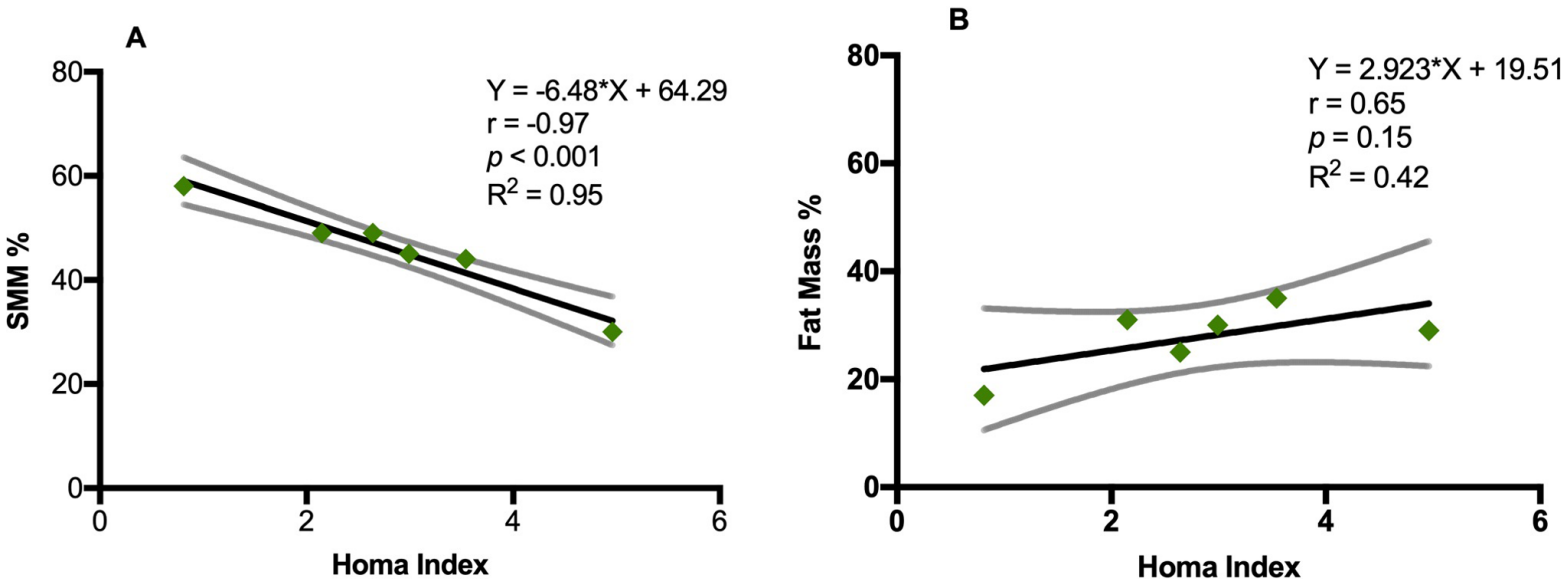
Correlation between Homa index and BIA variables in DM2 male group. **(A)** Pearson’s correlation between Homa index and SSM (%); **(B)** Pearson’s correlation between Homa index and fat mass (%). SSM: Skeletal muscle mass (%). Y= equation; r = Pearson’s correlation; R squared = coefficient of determination; *p* = p value.

Phase angle was below normal values in 56% of males and 89% of females (Figure 1). Compared to the healthy subjects, we found a significant reduction of PA in the DM2 group (DM2 PA mean ± SD 5.1 ± 1.2; HC PA mean ± SD 6.0 ± 1.1 *p* < 0.05). The male subgroup showed a higher reduction of FFM compared to females (78% and 22%, respectively), whereas both groups presented an increase in percentage of fat mass (56% of males, 22% of females) (Figure 2). There were no sex differences in BIA variables at Fisher’s exact test and at Student’s *t*-test, however we noted a higher relative risk of reduction of FFM in males and of PA in females, respectively (PA: 2 relative risk, 1–4.8 95% CI, *p* = 0.13; FFM 0.28 relative risk, 0.07–0.82 95% CI, *p* = 0.056; FM 0.8 relative risk, 0.3–2.03 95% CI, *p* = 0.9) (Figure 3). Moreover, we found a negative correlation between BMI and FFM (*r* − 0.64, *p* < 0.01) and a positive one with FM (*r* 0.64, *p* < 0.01). Vitamin D values showed a negative correlation with HOMA index (*r* − 0.41, *p* not significant) and BMI (*r* − 0.53, *p* = 0.05). Regarding gender-related BIA characteristics, we noted that only in the male group there was a positive correlation between HOMA index and FM (*r* 0.65, *R*^2^ 0.42, *p* = n.s.), and a negative correlation between HOMA index and SMM (*r* − 0.97, *R*^2^ 0.95, *p* < 0.001) (Figure 4).

### 3.3 Assessment of muscle strength, endurance and motor function (QMFT, 30CST, QMT)

**Figure 5:**
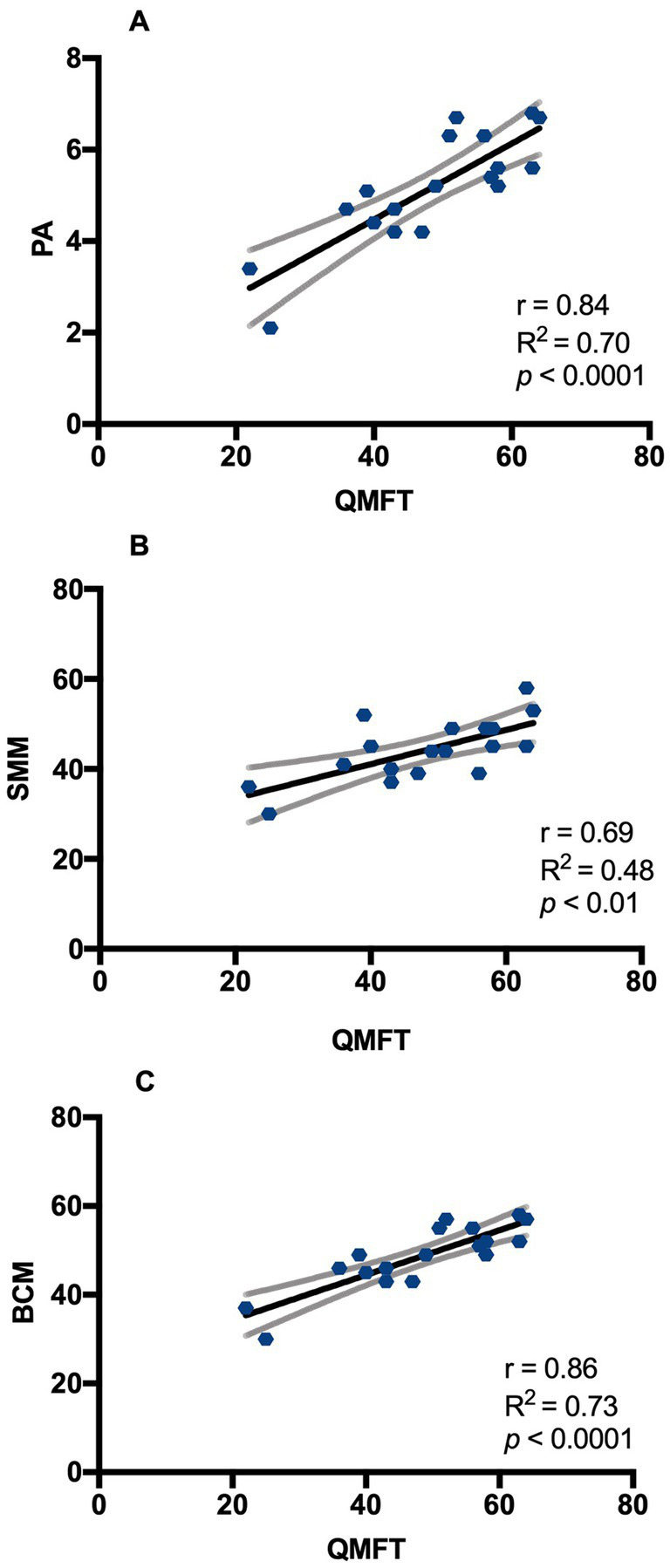
Correlation between QMFT and BIA parameters in DM2 patients. **(A)** Pearson’s correlation between QMFT and PA; **(B)** Pearson’s correlation between QMFT and SMM; **(C)** Pearson’s correlation between QMFT and BCM. QMFT: Quick motor function test; PA: Phase angle; SMM: Skeletal muscle mass (%); BCM: body cell mass (%). Y= equation; r = Pearson’s correlation; R squared = coefficient of determination; *p* = p value.

The mean value of the QMFT score was 48 ± 12.4 (mean ± SD) on a scale of 64, with no sex differences. At the 30SCT, 100% of males and 89% of females performed scores under normal values for gender and age (n.v. female ≥15 repetitions; n.v. male ≥17 repetitions). At QMT evaluation, the most frequently affected muscle group considering P5 values were elbow flexors for males and neck extensors for females (100% and 89% of patients respectively). Moreover, at least 90% of patients showed values below P50 for elbow, hip and neck flexors in males and for neck flexors and extensors, elbow flexors, hip flexors and knee extensors in females. The battery of functional motor tests we performed showed consistency between different tests. There was a positive correlation of QMFT with QMT sum score (*r* 0.54, *p* < 0.05) and with 30CST (*r* 0.67, *p* < 0.01). In turn, motor tests showed a good correlation with body composition parameters measured by BIA, as reported in Table 2. In particular, QMFT score had a direct correlation with Xc (*r* 0.78, *p* < 0.0001), PA (*r* 0.84, *p* < 0.0001), BCM (*r* 0.86, *p* < 0.0001), SMM (*r* 0.69, *p* < 0.01), SMMI (*r* 0.61, *p* < 0.01) and with BCMI (*r* 0.70, *p* = 0.001). Linear regression between QMFT and phase angle and BCM showed an *R*^2^ of 0.70 and 0.73, respectively (Figure 5).

**Table 2:** Correlation of motor assessments with BIA parameters.

	QMFT (*r*)	*p*	QMT sum score (*r*)	*p*	30CST (*r*)	*p*
R	0.16	n.s.	−0.29	n.s.	0.06	n.s.
Xc	0.77	***p* < 0001**	0.45	n.s.	0.49	***p* < 0.05**
PA	0.84	***p* < 0001**	0.60	***p* < 0.01**	0.58	***p* < 0.01**
FFM (%)	0.02	n.s.	0.04	n.s.	0.08	n.s.
FFMI (kg/h^2^)	−0.1	n.s.	0.3	n.s.	0.01	n.s.
SMM (%)	0.69	***p* < 0.01**	0.49	***p* < 0.05**	0.54	***p* < 0.05**
SMMI (kg/h^2^)	0.61	***p* < 0.01**	0.64	***p* < 0.01**	0.52	***p* < 0.05**
FM (%)	−0.02	n.s.	−0.04	n.s.	−0.08	n.s.
FMI (kg/h^2^)	−0.02	n.s.	0.06	n.s.	−0.01	n.s.
BCM (%)	0.86	***p* < 0001**	0.57	***p* < 0.05**	0.61	***p* < 0.001**
BCMI (kg/h^2^)	0.70	***p* < 0.001**	0.69	***p* < 0.001**	0.59	***p* < 0.01**

QMFT, quick motor function test; QMT, quantitative muscle testing; 30CST, 30s chair to stand test; R, resistance; Xc, reactance; PA, phase-angle; FFM, fat-free mass; FFMI, fat-free mass index; SMM, skeletal muscle mass; SMMI, skeletal muscle mass index; FM, fat mass; FMI, fat mass index; BCM, body cell mass; BCMI, body cell mass index.

Significant values are in bold.

n.s = not significant.

(r) = Pearson’s correlation.

## 4 Discussion

Myotonic dystrophy type 2 is characterized by slowly progressive proximal and axial muscle weakness, myotonia, and myalgia, and by other abnormalities such as insulin-insensitive type II diabetes mellitus and various endocrine alterations. Until recently, no disease-specific, sensitive, and validated outcome measures for DM2 were available. Lately, Montagnese et al. (5), proposed a panel of motor tests to evaluate the progression of the disease. The tests that better detected a weakness progression at 1 year were: QMT by HHD, QMFT and the Six minute walk test (5). Although the combination of these tests proved to be useful, their time-sensitivity in order to achieve a thorough insight in the pace of disease progression may need further investigation. Besides, it would be important to rely on an outcome measure or biomarker that can be used to monitor the natural history of the disease regardless of the degree of weakness. This is of paramount importance in order to achieve trial readiness. Recently, the evaluation of body composition changes has been implemented in clinical practice as an instrument to evaluate disease stage or as a prognostic tool. Indeed, reduction of FFM or lower values of PA at first evaluation are associated with a worse prognosis in several diseases, including neurological diseases (8–11). For instance, PA is a prognostic factor for survival in amyotrophic lateral sclerosis (16). In DM1, body composition, measured by dual-energy x-ray absorptiometry (DXA), showed significantly higher FM and lower FFM indexes compared to healthy subjects (17). Furthermore, a recent study demonstrated a good correlation of several BIA parameters with some measures of disease severity in DM1 (12). Therefore, we decided to assess BIA, together with a set of motor outcome measures, to evaluate its potential role in staging global muscle integrity in DM2 patients. In our cohort, there was a high prevalence of obesity, insulin resistance, metabolic syndrome, and vitamin D deficiency, in line with previous studies (18, 19). Vitamin D is known to influence muscle performance and to be regulated by adiposity. Although we could not find any correlation of vitamin D values with motor tests, a negative correlation with HOMA index and BMI was present. These data suggest that metabolism is seriously deranged and an alteration in values of body composition is very likely in such patients, and our findings with BIA confirm this hypothesis. First of all, the direct correlation of FM and the inverse correlation of FFM and SMM with BMI and WC corroborate the validity of BIA in detecting changes in body composition. Interestingly, BIA parameters were largely affected in patients: in particular, compared to normal values provided by the software, there was an increase in FM and a decrease in FFM and SMM, the latter more prevalent among males, similarly to what observed in DM1 (17, 20). A higher incidence of FFM reduction among males could be due to the influence of hypogonadism. In fact, reduced FFM and an excess of FM can be linked to an alteration of anti-Müllerian hormone, inhibin and estradiol, leading to a reduced synthesis of several myokines that exert an anabolic drive in muscles (21). Factors contributing to an increase of FM in DM2 should be further elucidated, although reduced resting energy expenditure might be involved, as in DM1 (17). On the other hand, mechanisms inducing a loss of lean body mass may be more straightforward, including insulin resistance, vitamin D deficiency, sedentary habits and, possibly, increased proteolysis as in DM1 (20). The inverse correlation between HOMA index and muscle mass in male DM2 patients may support the hypothesis that insulin resistance plays a role in the reduction of muscle mass in this disease. The major degree of muscle impairment in male patients may parallel the same gender-impact on strength reported in myotonic dystrophy type 1 (22). The reduction of PA we observed in almost half of male and nearly all female DM2 patients recalls similar findings in DM1 and may be regarded as a marker of deranged cell membrane function (12). Tests of muscle strength, endurance and motor function showed performances below normal ranges in general. Axial and proximal limb muscles resulted more affected than others, as expected in DM2. Interestingly, several BIA parameters showed a correlation with those of strength and motor tests. In particular, QMFT, QMT sum score and 30CST showed a fair to strong direct correlation with PA, SMM, SMMI, BCM and BCMI, which represent mainly muscle tissue. On the contrary, no correlation was seen with FFM, which includes bone tissue, and FM. These data suggest that a subset of BIA parameters are faithful markers of the degree of muscle impairment in DM2. These findings are similar to those obtained in DM1 patients, where a direct correlation of PA and other parameters with clinical muscle involvement was demonstrated (12).

The present study has several limitations, one of them being the small dimension of the patients’ sample, which is due to the rarity of the disease, corresponding to a prevalence rate of 1:100,000 in Italy and to the involvement of a single center, this being a pilot study (4). In addition, the cross-sectional nature of the study does not allow acquiring data on the progression of the disease. It would be interesting to extend our present observations with a longitudinal protocol applied to a larger cohort in order to explore the sensitivity of BIA to the slow progression of DM2. Further studies could be conducted on patients with DM2 to evaluate variation between pre- and post-nutritional therapy status, as already demonstrated for cancer patients (23). We are also aware that the gold standard to measure body composition is represented by DXA, however, this is a more invasive technique as compared to BIA and would not be a first line tool in longitudinal studies requiring multiple testing. On the other hand, a significant correlation between BIA and DXA-derived data on body composition have been detected by some authors (24, 25).

In conclusion, the present study shows that whole-body BIA may be a useful method to monitor the severity of muscle wasting and impairment in DM2. These preliminary data need confirmation in an extended, multicenter study in a broader sample. The different degree of BIA alterations between genders may reflect a sex-related involvement of lean body mass in this disease. The potential of this technique to capture changes during the slow progression of DM2 should be tested in longitudinal studies, keeping in mind this is an essential goal to be reached while getting ready for future therapeutic trials.

## Data Availability

The raw data supporting the conclusions of this article will be made available by the authors, without undue reservation.
